# Determining antimicrobial susceptibility in *Salmonella enterica* serovar Typhimurium through whole genome sequencing: a comparison against multiple phenotypic susceptibility testing methods

**DOI:** 10.1186/s12866-019-1520-9

**Published:** 2019-07-02

**Authors:** Nana Mensah, Yue Tang, Shaun Cawthraw, Manal AbuOun, Jackie Fenner, Nicholas R. Thomson, Alison E. Mather, Liljana Petrovska-Holmes

**Affiliations:** 1 Animal and Plant Health Agency, Weybridge, Addlestone, Surrey, UK; 2 The Wellcome Trust Sanger Institute, Hinxton, Cambridgeshire, UK; 3 University of Cambridge, Cambridge, Cambridgeshire, UK; 4 Present Address: Quadram Institute Bioscience, Norwich, Norfolk, UK; 5 University of East Anglia, Norwich, Norfolk, UK

**Keywords:** Antimicrobial susceptibility tests, Whole genome sequencing, Disk diffusion, Agar dilution, Broth microdilution, *Salmonella enterica* serovar typhimurium

## Abstract

**Background:**

UK public health organisations perform routine antimicrobial susceptibility tests (ASTs) to characterise the potential for antimicrobial resistance in *Salmonella enterica* serovars. Genetic determinants of these resistance mechanisms are detectable by whole genome sequencing (WGS), however the viability of WGS-based genotyping as an alternative resistance screening tool remains uncertain. We compared WGS-based genotyping, disk diffusion and agar dilution to the broth microdilution reference AST for 102 *Salmonella enterica* serovar Typhimurium (*S. Typhimurium*) isolates across 11 antimicrobial compounds.

**Results:**

Genotyping concordance, interpreted using epidemiological cut-offs (ECOFFs), was 89.8% (1007/1122) with 0.83 sensitivity and 0.96 specificity. For seven antimicrobials interpreted using *Salmonella* clinical breakpoints, genotyping produced 0.84 sensitivity and 0.88 specificity. Although less accurate than disk diffusion (0.94 sensitivity, 0.93 specificity) and agar dilution (0.83 sensitivity, 0.98 specificity), genotyping performance improved to 0.89 sensitivity and 0.97 specificity when two antimicrobials with relatively high very major error rates were excluded (streptomycin and sulfamethoxazole).

**Conclusions:**

An 89.8% concordance from WGS-based AST predictions using ECOFF interpretations suggest that WGS would serve as an effective screening tool for the tracking of antimicrobial resistance mechanisms in *S.* Typhimurium. For use as a standalone clinical diagnostic screen, further work is required to reduce the error rates for specific antimicrobials.

**Electronic supplementary material:**

The online version of this article (10.1186/s12866-019-1520-9) contains supplementary material, which is available to authorized users.

## Background

Antimicrobial resistance (AMR) is a global health emergency. In the EU alone, drug-resistant infections result in healthcare costs of at least €1.5 billion per year [1, 2]. The ability to rapidly acquire genetic elements conferring resistance to antimicrobial drugs from the environment contributes to the survivability of infectious foodborne pathogens. Among these, *Salmonella enterica* serovar Typhimurium (*S.* Typhimurium) is one of the most common sub-species isolated from livestock in the UK [3]. In humans, drug-resistant *S*. Typhimurium has been associated with a higher risk of infection, frequency of hospitalization, illness and risk of death than pan-susceptible strains [1]. Accurate, rapid and cost-effective classification of AMR in infectious agents such as *S*. Typhimurium is therefore critical to managing disease burden.

The broth microdilution antimicrobial susceptibility test (AST) is defined in the International Standards Organisation standard 20,776–1:2006 [4] as the reference method for testing the in vitro activity of antimicrobial agents against infectious bacteria. This test determines the minimum inhibitory concentration (MIC) of an antimicrobial required to limit bacterial growth. Although broth microdilution is the mandated method for reporting AMR surveillance in the EU harmonised surveillance programme, many research and surveillance laboratories maintain the use of legacy reference AST methods. For example, resistance in bacterial isolates submitted to UK veterinary laboratories is measured using the disk diffusion AST [5]. Phenotypic ASTs determine the viability of bacteria in response to antimicrobial exposure and are therefore unable to distinguish between the genetic mechanisms responsible. At most, the cumulative effect of underlying AMR mechanisms may be inferred from ASTs using interpretive criteria. For this purpose, whole genome sequencing (WGS) is a high-resolution assay capable of identifying molecular AMR mechanisms in *S.* Typhimurium and other bacteria [6–8]. Developments in next generation sequencing technologies have led to a steady decrease in the cost of WGS; WGS has been performed for as little as £40 per bacterial genome and in the past decade, this and the increasing availability of the technology has been reflected in the greater than 100-fold increase in publicly available genomes [9, 10]. In a single assay, WGS is able to facilitate subspecies typing, AMR prediction and phylogenetic source attribution, prompting international efforts to validate WGS informatics pipelines for surveillance applications [11].

Previous assessments of *Salmonella* AMR identified through WGS have predominantly used a single phenotypic AST method in comparisons, such as disk diffusion [12, 13], agar dilution [14] and broth microdilution [6]. To determine robustly the utility of WGS for AMR prediction, comparisons against multiple phenotypic tests representing clinical and veterinary screening environments are required. Whereas ASTs interpreted using clinical breakpoints inform clinicians of the potential for antimicrobial treatment failure, epidemiological cut-offs (ECOFFs) dictate the upper-limit MIC for isolates devoid of acquired resistance mechanisms as determined by a species-specific MIC population distribution. To facilitate meta-analysis, the European Committee on Antimicrobial Susceptibility testing (EUCAST) subcommittee recommends ECOFFs as the primary interpretive criteria for WGS comparisons against phenotypic ASTs, along with secondary analyses using clinical breakpoints on the same data set [15]. This study contributes to the growing evidence base assessing WGS for bacterial AMR determinations by comparing *S*. Typhimurium genotyping predictions as assessed by WGS to the broth microdilution reference method using both interpretive criteria. Additionally, agar dilution and disk diffusion ASTs are compared to determine the utility of WGS against these alternative screening methods.

## Methods

### Isolate selection

One hundred and two S. Typhimurium isolates, isolated between 1992 and 2012, were cultured from the Animal and Plant Health Agency (APHA, Addlestone, UK) strain collection. Duplicate strains were not identified or excluded, however the selection criteria involved randomised selection of isolates; a minimum of two per year across a 20 year timeframe. At the time of submission, all isolates were serotyped in accordance with the White-Kauffman-Le Minor scheme [16] and phenotypic AMR profiles were determined by disk diffusion. Selection criteria therefore included a minimum of two isolates per year and a range of overlapping phenotypic AMR profiles. Additional quality controls were provided by repeated serotyping and disk diffusion testing to confirm the phenotype of the selected strains.

### Antimicrobial susceptibility testing

Reference broth microdilution MICs were obtained using the Sensititre™ Complete Automated System with EUVSEC and CMV3AGNF panels (Trek Diagnostic Systems, West Sussex, UK). ECOFFs published by EUCAST [17] were applied to these results. In the cases of sulfamethoxazole and sulfisoxazole, ECOFFs could not be established from EUCAST data therefore the >256 μg/mL ECOFF used in the EFSA/ECDC and NARMS monitoring programs was applied [18, 19]. Wild-type (WT) and non-wild-type (NWT) resistance profiles were determined for 11 antimicrobials, representing a subset of those historically evaluated in *Salmonella* AMR screening at the APHA: ampicillin (Amp), chloramphenicol (Chl), ciprofloxacin (Cip), gentamicin (Gen), nalidixic acid (Nal), streptomycin (Str), sulfamethoxazole (Smx), trimethoprim/sulfamethoxazole (1:19) (Sxt), sulfisoxazole (Sul), tetracycline (Tet) and trimethoprim (Tri).

Seven antimicrobials were selected from this initial list for resistance profiling by disk diffusion and agar dilution ASTs. This selection was made based on the available clinical breakpoints for both phenotyping methods. Antimicrobial disks (Thermo-Fisher Scientific Ltd) and antimicrobial powders (Sigma-Aldrich) containing ampicillin, chloramphenicol, gentamicin, nalidixic acid, streptomycin, sulfamethoxazole and trimethoprim/sulfamethoxazole (1:19) were used to determine clinical resistance profiles. Specifically, disk diffusion tests were conducted on Isosensitest Agar (Oxoid) following the Kirby-Bauer method described by the British Society for Antimicrobial Chemotherapy (BSAC) [20]. Although legacy zone diameter readings informed isolate selection, disk diffusion testing was repeated for this study to account for temporal factors in comparisons with phenotypic and genotypic ASTs. Isolates were tested by agar dilution using the method described by Wiegand et al. [21]. Zone diameters and MICs determined by all phenotypic methods were interpreted using EUCAST clinical breakpoints [22], with the following exceptions: breakpoints for nalidixic acid and sulfonamides have not been determined by EUCAST, therefore breakpoints published by the Clinical and Laboratory Standards Institute were applied [23]; although no longer used clinically, the legacy NARMS 64 μg/mL streptomycin clinical breakpoint was used as in previous studies [6]. Control strains *E. coli* ACTC25922 and *E. coli* NCTC10418 were included for susceptibility test validation. ECOFFs, clinical breakpoints, dilution ranges and disk contents used are presented in Additional file 1: Table S1. All phenotyping results are presented in Additional file 1: Table S2.

### Whole genome sequencing and AMR genotyping

Bacterial DNA was extracted with the MagNA Pure LC DNA Isolation Kit III (Roche) according to manufacturer’s instructions and sequenced on the Illumina HiSeq platform producing paired-end (2x125bp) reads. Acquired resistance determinants were identified from WGS reads using ARIBA [24] in combination with the Resfinder v3.0 database [25]. Additionally, the presence of point mutations associated with AMR was determined using Pointfinder v3.0 [26]. Whole genome sequences of the 102 *S*. Typhimurium isolates were submitted to the European Nucleotide Archive under the study accession PRJEB10999. WGS AMR genotyping profiles and AMR gene accession numbers are presented in Additional file 1: Table S3. Accession numbers for genes identified in the *Salmonella* genomes are presented in Additional file 1: Table S4 along with their observed frequency including full matches, partial, fragmented and interrupted assemblies.

### Genotype-phenotype comparisons

Broth microdilution MICs interpreted with ECOFFs are classified as wild-type (susceptible) or non-wild-type (resistant), whereas phenotypic tests interpreted using clinical breakpoints are classified as resistant or sensitive. ASTs with ‘intermediate’ interpretation results are classified as sensitive in this study. Resistant or non-wild-type WGS genotypes are defined by the presence of one or more resistance determinants for a tested antimicrobial. Identified genes were matched to their corresponding phenotypes using the schema presented in Additional file 1: Table S4. Very Major Error (VME) and Major Error (ME) classifications were assigned to genotypic and phenotypic test results based on concordance with the broth microdilution reference. A VME is defined by a sensitive or wild-type AST prediction coupled with a resistant or non-wild-type reference phenotype. Similarly, MEs are defined by a resistant or non-wild-type AST prediction with a sensitive or wild-type reference phenotype. Error rates are presented as the percentage of VMEs or MEs over the total number of isolates with the corresponding reference phenotype. Sensitivity, specificity, positive predictive values, negative predictive values and 95% confidence intervals (CI) were calculated using the R bdpv package [27]. Analysis scripts are available online at: https://github.com/NMNS93/Mensah_SalmonellaWGS.

## Results

### Genotype-phenotype comparison: epidemiological cut-off criteria

Genotyping was concordant with 1007/1122 (89.8%) broth microdilution AST results interpreted with ECOFFs (Table 1). One hundred and fifteen discrepancies were observed, resulting in a 4.5% ME rate (*n* = 28) and a 17% VME rate (*n* = 87). Fifty-one (59%) VMEs were the result of discordant sulfisoxazole and sulfamethoxazole predictions. Similarly, genotyping produced VMEs for all eight gentamicin non-wild-type isolates. WGS AMR predictions granted 0.83 sensitivity and 0.96 specificity (95%CI 0.94–0.97) overall (Table 2). Excluding the results for sulfamethoxazole and sulfisoxazole ASTs, which presented consistently high VMEs, genotyping was concordant with 857 (93.4%) ASTs. For this subset excluding sulfamethoxazole and sulfisoxazole, genotyping presented 0.89 sensitivity and 0.96 specificity.

**Table 1:** Comparison of WGS-based genotyping and phenotypic ASTs against the broth microdilution reference. Frequency of isolates assigned to classification groups after phenotypic testing (broth microdilution) and application of interpretive criteria (epidemiological cut-off and clinical breakpoint). Genotype concordance with classification groups is provided for each interpretive criterion. Antimicrobials with ‘-‘values were not assessed due to the absence of established breakpoints or susceptibility test data

Antimicrobial	Epidemiological cut-off			Clinical breakpoint		
	Non-Wild Type	Wild Type	Genotype concordance (%)	Resistant	Sensitive	Genotype Concordance (%)
Ampicillin	57	45	95.10	57	45	95.10
Chloramphenicol	49	53	94.12	49	53	94.12
Ciprofloxacin	27	75	96.08	-	-	-
Gentamicin	8	94	92.16	7	95	93.14
Nalidixic Acid	28	74	95.10	28	74	95.10
Sulfamethoxazole	69	33	76.47	69	33	76.47
Streptomycin	66	36	89.22	26	76	55.88
Sulfisoxazole	73	29	70.59	-	-	-
Trimethoprim-Sulfamethoxazole (1:19)	33	69	93.14	30	72	96.08
Tetracycline	57	45	89.22	-	-	-
Trimethoprim	32	70	96.08	-	-	-
Overall	499	623	89.75	266	448	86.55

**Table 2:** Sensitivity and specificity of WGS-based antimicrobial resistance predictions against the broth microdilution reference AST. Epidemiological cut-offs are applied as interpretive criteria for the reference AST

Antimicrobial	Genotyping sensitivity (95% CI)	Genotyping specificity (95% CI)
Ampicillin	0.96 (0.88–1)	0.93 (0.82–0.99)
Chloramphenicol	0.94 (0.83–0.99)	0.94 (0.84–0.99)
Ciprofloxacin	0.85 (0.66–0.96)	1 (0.95–1)
Gentamicin	0 (0–0.37)	1 (0.96–1)
Nalidixic Acid	0.82 (0.63–0.94)	1 (0.95–1)
Sulfamethoxazole	0.67 (0.54–0.78)	0.97 (0.84–1)
Streptomycin	0.92 (0.83–0.97)	0.83 (0.67–0.94)
Sulfisoxazole	0.62 (0.5–0.73)	0.93 (0.77–0.99)
Trimethoprim-Sulfamethoxazole	0.88 (0.72–0.97)	0.96 (0.88–0.99)
Tetracycline	0.95 (0.85–0.99)	0.82 (0.68–0.92)
Trimethoprim	0.94 (0.79–0.99)	0.97 (0.9–1)
Overall	0.83 (0.79–0.86)	0.96 (0.94–0.97)

### Genotype-phenotype comparison: clinical breakpoint criteria

In total, 714 broth microdilution AST results were interpreted with clinical breakpoints, representing ASTs for seven antimicrobials. Genotyping was concordant with 618 (86.6%) broth microdilution reference ASTs, which was fewer than the 661 (92.6%) agar dilution and 665 (93.1%) disk diffusion results concordant with the reference (Table 1). Agar dilution produced the highest VME rate of 17% (*n* = 45) followed by 16% for genotyping (*n* = 43) and 6.93% for disk diffusion (*n* = 17) (Table 3). The agar dilution VMEs were split between chloramphenicol (*n* = 14) and streptomycin (*n* = 16). The majority of genotyping VMEs resulted from sulfamethoxazole resistance predictions (*n* = 23), as found using the ECOFFs. ME rates were highest for genotyping at 12% (*n* = 53), followed by 7.14% disk diffusion (*n* = 32) and 1.79% for agar dilution (*n* = 8). Streptomycin resistance predictions were responsible for 43/53 genotyping MEs and 31/32 disk diffusion MEs. WGS genotyping sensitivity was 0.84 (95%CI 0.79–0.88); this was lower than disk diffusion (0.94 sensitivity, 95%CI 0.90–0.96) yet higher than agar dilution (0.83 sensitivity, 95%CI 0.78–0.87). In contrast, agar dilution demonstrated the highest specificity of 0.98 (95%CI 0.97–0.99) followed by disk diffusion (0.93, 95%CI 0.90–0.95) and genotyping (0.88, 95%CI 0.85–0.91) (Table 3). Excluding ASTs with high error rates (streptomycin and sulfamethoxazole) resulted in improved genotyping results of 0.89 sensitivity (95%CI 0.83–0.94), 0.97 specificity (95%CI 0.95–0.99) and 94.7% concordance (483/510) for the remaining five antimicrobials.

**Table 3:** Sensitivity and specificity of phenotypic ASTs and genotypic AMR predictions against the broth microdilution reference AST. Clinical breakpoints are applied as interpretive criteria for all ASTs

Antimicrobial	Sensitivity (95% CI)			Specificity (95% CI)		
	Disk diffusion	Agar dilution	Genotyping	Disk diffusion	Agar dilution	Genotyping
Ampicillin	0.95 (0.85–0.99)	0.95 (0.85–0.99)	0.96 (0.88–1)	1 (0.92–1)	1 (0.92–1)	0.93 (0.82–0.99)
Chloramphenicol	0.92 (0.8–0.98)	0.71 (0.57–0.83)	0.94 (0.83–0.99)	0.98 (0.9–1)	0.98 (0.9–1)	0.94 (0.84–0.99)
Gentamicin	0.71 (0.29–0.96)	0.86 (0.42–1)	0 (0–0.41)	1 (0.96–1)	1 (0.96–1)	1 (0.96–1)
Nalidixic Acid	0.82 (0.63–0.94)	0.75 (0.55–0.89)	0.82 (0.63–0.94)	1 (0.95–1)	0.96 (0.89–0.99)	1 (0.95–1)
Sulfamethoxazole	0.97 (0.9–1)	0.97 (0.9–1)	0.67 (0.54–0.78)	1 (0.89–1)	0.94 (0.8–0.99)	0.97 (0.84–1)
Streptomycin	0.96 (0.8–1)	0.38 (0.2–0.59)	0.92 (0.75–0.99)	0.59 (0.47–0.7)	1 (0.95–1)	0.43 (0.32–0.55)
Trimethoprim-Sulfamethoxazole	1 (0.88–1)	0.93 (0.78–0.99)	0.97 (0.83–1)	1 (0.95–1)	0.97 (0.9–1)	0.96 (0.88–0.99)
Overall	0.94 (0.9–0.96)	0.83 (0.78–0.87)	0.84 (0.79–0.88)	0.93 (0.9–0.95)	0.98 (0.97–0.99)	0.88 (0.85–0.91)

### Aminoglycoside resistance

**Fig. 1:**
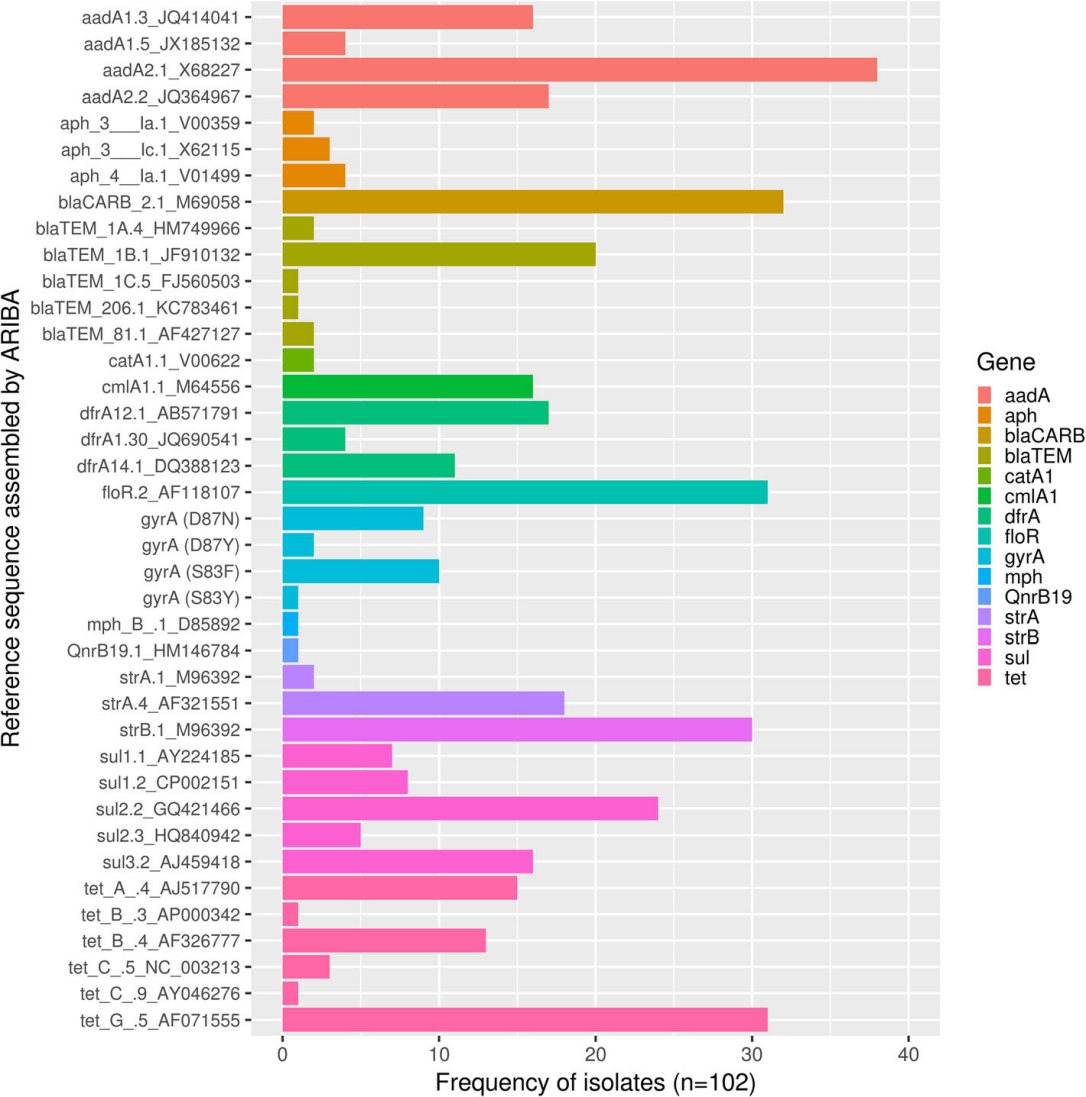
Frequency of *Salmonella enterica* serovar Typhimurium isolates identified with each reference sequence. Listed reference sequences are putative resistance genes fully assembled from isolate genomes by ARIBA

Streptomycin resistance determinants, *aadA* (*n* = 60) or *strAB (n = 29),* were identified in 67 (65.6%) isolates (Fig. 1 and Additional file 1: Table S4) resulting in 89% genotyping concordance with ECOFF interpretations. Resistance determinants were detected in 6/16 streptomycin wild-type isolates, resulting in MEs (MICs 16 μg/ml; S02606–01, S00795–02, S03632–02, L00777–11, S02636–11, S02347–12). Genotyping was 55.8% concordant with streptomycin clinical breakpoints as 43/76 streptomycin sensitive isolates (MIC ≤64 μg/ml) contained complete resistance gene hits. AMR determinants were not found for resistant isolates S02185–06 and L02428–05, although *aadA1* (X68227) was partially assembled in the latter.

Gentamicin resistance determinants were not identified in the *S*. Typhimurium genomes. However, seven isolates with non-wild-type and clinical gentamicin resistance (S10543–93, S01710–04, S04488–04, L00746–11, S03924–11, S06636–12 and S00478–13) had a partially assembled *aac3-IVa* gene (X01385). An additional NWT strain (L02428–05) contained this partial assembly and presented a lower MIC at one doubling dilution above the 2 μg/ml gentamicin ECOFF.

### Beta-lactam resistance

Fifty-eight isolates (56.8%) contained either *bla*_*TEM*_ (*n* = 26) or *bla*_*CARB*_ (*n* = 32) (Fig. 1 and Additional file 1: Table S4) conferring ampicillin resistance. Concordance using both ECOFFs and clinical breakpoints was 95%, with two VMEs (S04635–04, S04655–09; MIC > 64 μg/ml) and only three MEs (S02606–01, L00777–11, L01210–11; MIC ≤2 μg/ml). MEs were due to genotype hits for *bla*_*CARB-2*_ (M690858), *blaTEM-1B* (JF910132) and *bla*_*TEM-206*_ (KC783461) respectively.

### Phenicol resistance

Phenicol resistance determinants *floR* (*n* = 31), *cmlA1* (*n* = 16) and *catA1* (n = 2) were present in 46 isolates (45%) (Fig. 1 and Additional file 1: Table S4). All chloramphenicol non-wild-type isolates were also classified as resistant using clinical breakpoints. Genotyping identified chloramphenicol AMR genes in 46/49 R/NWT isolates, and 3/53 S/WT isolates, resulting in 94% AMR prediction concordance for this antimicrobial using both interpretive criteria. Three isolates with MEs (S10423–92, *catA1* (V00622); S02606–01, *floR* (AF118107); L00777–11, *cmlA* (M64556)) had MICs at the 8 μg/ml clinical breakpoint, one doubling dilution from the 16 μg/ml ECOFF. Of three isolates with VMEs (S06618–99, S01117–00, S04635–04), a partial *floR* gene was assembled from one genome (S04635–04).

### Quinolone resistance

Twenty-two isolates harboured point mutations in the quinolone resistance determining region of *gyrA*: Ser83-Phe (*n* = 10), Ser83-Tyr (*n* = 1), Asp87-Asn (*n* = 9) and Asp87-Try (*n* = 2) (Fig. 1 and Additional file 1: Table S4). No other known *S.* Typhimurium quinolone resistance mutations were detected. Additionally, isolate S06001–08 contained the plasmid-mediated quinolone resistance gene *qnrB19* (HM146784). Isolates classified as nalidixic acid resistant using clinical breakpoints were also classified as non-wild-type using ECOFFs, therefore genotypic resistance determinants showed 95% concordance for both interpretive criteria. These discrepancies resulted from a lack of nalidixic acid resistance determinants in five isolates with MICs > 128 μg/ml (S01117–00, S02606–01, S04635–04, S04655–09, S06636–12). Genotype-phenotype correlations for ciprofloxacin mirrored nalidixic acid results using ECOFFs except in the case of S6636–12, which granted one fewer VME due to a 0.03 μg/ml WT ciprofloxacin MIC.

### Sulfonamide and trimethoprim resistance

Sulfonamide resistance determinants *sul1* (*n* = 15), *sul2* (*n* = 29) and *sul3* (*n* = 16) were present in 47 isolates (46.1%) (Fig. 1 and Additional file 1: Table S4). For sulfamethoxazole reference ASTs, all isolates classified as resistant using clinical breakpoints were also classified as non-wild-type using ECOFFs. Sulfamethoxazole AMR gene presence resulted in 76% concordance for both interpretive criteria, resulting from 23 VMEs and 1 ME (L00777–11; MIC 64 μg/ml; *sul2* (GQ421466), *sul3* (AJ459418)). For sulfisoxazole reference ASTs interpreted with ECOFFs, *sul* genes presence produced 71% genotyping concordance. Twenty-eight isolates had genotyping VMEs. Two *sul* wild-type isolates had MEs: L00777–11 with *sul2* and *sul3* and S02347–12 with *sul2*.

Resistance to folate synthesis inhibitors containing trimethoprim was predicted by the presence of *dfrA* gene variants, which were identified in 32 isolates (*dfrA1* (*n* = 4), *dfrA12* (*n* = 17) and *dfrA14* (*n* = 11)). Trimethoprim AST results were classified using the > 2 μg/ml ECOFF and the resulting genotype concordance was 96% for WT and NWT ASTs. Two MEs were detected in isolates with 0.25 μg/ml trimethoprim MICs: S00285–01 with *dfrA14* (DQ388123) and L00777–11 with *dfrA12* (AB571791). Classifications for the sulfamethoxazole-trimethoprim compound (SXT) differed between interpretive criteria, producing different genotyping correlations. Genotyping concordance was 93% for SXT interpreted with the 1 μg/ml ECOFF and 96% for SXT interpreted with the 4 μg/ml clinical breakpoint. MEs for both interpretive criteria were the result of genotype predictions for the same three isolates (*dfrA14* (DQ388123) in S00285–01; *dfrA12* (AB571791) in S01710–04 and L00777–11). Whereas genotyping predictions compared with SXT clinical breakpoint interpretations produced only one VME (S02202–01), predictions compared with SXT ECOFF interpretations produced four (S02202–01, S07053–03, S04635–04, S04055–07).

### Tetracycline resistance

Sixty-two isolates harboured tetracycline resistance determinants *tetA* (*n* = 15), *tetB* (*n* = 14), *tetC* (*n* = 4) and *tetG* (*n* = 31) (Fig. 1 and Additional file 1: Table S4). Genotyping concordance was 89% using the tetracycline > 8 μg/ml ECOFF for interpretation. MEs were found in eight isolates with tetracycline MICs ≤4 μg/ml (S10423–92, S02202–01, S02606–01, S03249–01, S00795–02, S06216–03, L00777–11 and S02347–12). Discrepancies resulted from hits against *tetA* (AJ517790; *n* = 4), *tetG* (AF071555; *n* = 3) and *tetB* (AF326777; *n* = 1). Three isolates with VMEs had tetracycline MICs ≥32 μg/ml (S01117–00, S02357–04 and S04635–04).

## Discussion

Comparisons of AMR genotyping methods to phenotypic ASTs are essential to determine the true utility of WGS for AMR surveillance. This study contributes to the growing body of evidence by providing genotype-phenotype comparisons for both epidemiological and clinical contexts, and in comparing multiple phenotypic ASTs. *S*. Typhimurium AMR genotypes were 89.8% concordant for ASTs interpreted with ECOFFs across 11 antimicrobials. Clinical breakpoints were available for seven of these antimicrobials and after applying these criteria to the same broth microdilution dataset, WGS concordance was 86.6%. A further comparison demonstrated that agar dilution and disk diffusion presented more favourable results than genotyping with 92.6 and 93.1% concordance respectively (Table 3). Genotyping predictive errors were more common for specific antimicrobials. For example, excluding sulfonamides in ECOFF interpretations raised concordance to 93.4%. Genotyping concordance was noticeably lower for sulfamethoxazole ASTs (76%) than the trimethoprim-sulfamethoxazole compound (93%) ASTs. However, this only reflects the predictive accuracy of different AMR genes identified as sulfonamides and trimethoprim inhibit enzymes at disparate stages of the folate synthesis pathway, through different molecular mechanisms [28]. Similarly for clinical breakpoint interpretations, excluding sulfamethoxazole and streptomycin phenotypes raised concordance to 94.7%. This demonstrates that genotyping predictions may be more accurate than agar dilution and disk diffusion ASTs for specific antimicrobial classes.

Interestingly, 44/96 clinical breakpoint errors and 53/115 ECOFF errors occurred where isolate MICs were within 1 doubling dilution of the classification breakpoint. Interpretive criteria for clinical breakpoints typically include ‘intermediate’ classification ranges that reflect the concentration range for which isolates contain resistance determinants but do not present MICs past the resistance threshold. In this analysis, intermediates were classed as susceptible, therefore isolates in this range may contribute to these errors despite otherwise accurate genotyping calls. Such increases in error frequency near MIC breakpoints were especially true for streptomycin comparisons, where 39/45 mismatches were within 1 dilution of the clinical breakpoint. Misclassification of streptomycin resistance by WGS genotyping has been well documented in enterobacteria, including *Salmonella* and *E. coli* spp. [6–8, 29–31]. These mismatches have been attributed to insufficient breakpoints and gene inactivation mechanisms. For example, environmental and mutational activation of the cryptic aadA gene can increase expression of streptomycin resistance in *Salmonella enterica* [32]. We applied the ≥64μg/ml breakpoint used by McDermott et al. (2016) [6] to identify highly-resistant isolates. Both CLSI and EUCAST interpretation tables note that for *Salmonella spp.*, aminoglycosides may appear active in vitro but are not effective clinically and should not be reported as susceptible. Therefore, a lack of clinical use and difficulty in classification contributes to the absence of clinical breakpoints for streptomycin. Tyson et al. (2016, [33]) suggest incorporating genotypic resistance determinants into predictions of streptomycin breakpoints and such refinements to interpretive criteria may improve genotyping prediction accuracy against.

A number of genotyping predictive errors were accompanied by interrupted, fragmented or partial assemblies of genes identified using ARIBA. As these do not constitute complete genes, they were not reported for these isolates. All gentamicin non-wild-type (*n* = 8) isolates were inaccurately classified, however an interrupted *aac3-IVa* gene (X01385) was identified by ARIBA in 7/8 non-wild-type isolates, which were also resistant above the gentamicin clinical breakpoint. In addition, we acknowledge that the low gentamicin resistance prevalence affects the reliability of genotyping specificity estimates. In 22 isolates with sulfonamide VME mismatches, fragmented *sul1* genes (CP002151 or EU855787) were identified by ARIBA. Resistance mechanisms may therefore have variable detection rates dependent on factors such as sequencing quality and genomic arrangement.

Multiple studies have reported that resistomes determined by WGS show a high correlation with clinical resistance by broth microdilution ASTs in Gram-negative bacteria. McDermott et al. (2016) observed a genotype-phenotype correlation of 99% in 640 *Salmonella* [6] and more recently, Neuert et al. identified genotyping discrepancies in only 0.17% of isolate-antimicrobial combinations [14]. Similarly, Tyson et al. report a 98.5% overall correlation in 76 *E. coli* [8] and Zhao et al. found a 99.2% correlation in 114 *Campylobacter* isolates [34]. Including these studies, sensitivity and specificity of WGS-based AMR predictions against ASTs in a range of bacterial species has been reported at ≥96%. [9, 35, 36]. Previous studies also quote low WGS susceptibility testing error rates [35, 37] within the bounds of those deemed tolerable by the US FDA susceptibility testing devices (< 1.5 confidence range limit for VMEs and < 3.0% for MEs, with a minimum category agreement of > 89.9%)[38]. We observed lower levels of category agreement and higher error rates between WGS genotypes and *S*. Typhimurium phenotypes than previously reported. However, this study identifies a new lower-bound genotyping accuracy range for specific antimicrobials and provides a comparison with alternative phenotypic methods. Additionally, several studies present genotype-phenotype comparisons using in-house scripts and unpublished AMR gene databases from which results cannot be reproduced. Here, we leverage the open-source bioinformatics algorithm ARIBA along with the Resfinder and Pointfinder databases. ARIBA has the advantage of a local assembly method that accurately determines interrupted or partially present genes from sequence data. Data pre-processing and analysis procedures are provided as R scripts.

The observed misclassifications point to the current limits of WGS-based genotyping. Genotyping approaches require AMR sequence databases containing known resistance genes. Although WGS may accurately sequence novel genetic mechanisms, clinically resistant isolates may be falsely labelled as sensitive if these mechanisms are not represented in AMR databases. Additionally, WGS-based genotyping methods used in this study are unable to account for mechanisms that attenuate or inhibit gene expression. At the molecular level this includes factors such as the proximity of class 1 integron promoter regions, the copy number of gene cassettes and the potency of primary and secondary promoters [30, 39, 40]. The limitations described are inherent to the challenge of predicting AMR from the presence of known resistance genes alone.

The current evidence base for using WGS to infer antimicrobial susceptibility requires significant expansion [15]. Alternatives to the reference broth microdilution AST remain relevant to diagnostic and surveillance laboratories worldwide, therefore the inclusion of agar dilution and disk diffusion methods in this study provides context for these institutions to evaluate the use of WGS to predict *S. Typhimurium* AMR susceptibility. Over time the increasing predictive power of computational analysis, coupled with decreasing sequencing costs, will make WGS a more viable AMR screening tool for laboratories worldwide. Cost-benefit assessments should factor in the extended utility of WGS beyond AMR screening. WGS presents a powerful assay for a genotype-driven approach to epidemiology, capable of detecting AMR genes, clonal and plasmid typing, detecting mechanisms for gene transfer and virulence, and the source attribution of novel isolates. Future work should identify the role of downstream events that reduce susceptibility in discordant predictions where gene presence does not account for observed phenotypes. As further replicative and investigative studies identify new resistance mechanisms and improve the reliability of AMR sequence databases, a decrease in error rates may see this technical method supplement, and potentially supersede, established assays.

## Conclusion

We compared WGS for AMR prediction against 3 phenotypic tests including disk diffusion, agar dilution and the gold standard broth microdilution representing clinical and veterinary screening environments. The European Committee on Antimicrobial Susceptibility testing (EUCAST) subcommittee recommends ECOFFs as the primary interpretive criteria for WGS comparisons against phenotypic ASTs, along with secondary analyses using clinical breakpoints. Genotypic predictions correctly characterised isolates as wild-type or non-wild-type for 89.8% (1007/1122) of phenotypic assays with 0.83 sensitivity and 0.96 specificity using broth microdilution as the reference susceptibility test method. Genotyping was comparable with disk diffusion (sensitivity 0.94, specificity 0.93) and agar dilution (sensitivity 0.83, specificity 0.98) resistance phenotypes where antimicrobials with high very major error rates were excluded, producing 0.89 sensitivity and 0.97 specificity. Results from this study indicate that with further work to reduce the error rates for specific antimicrobials, WGS would serve as an effective screening tool for the tracking of AMR mechanisms in *S.* Typhimurium.

## Additional file

Additional file 1: This file contains four Microsoft Excel Sheets with additional information. **Table S1.** describes the interpretation criteria, dilution ranges and antimicrobial disk contents used for phenotypic susceptibility testing. **Table S2.** presents the results of phenotypic susceptibility tests and whole genome sequence genotyping. Classification categories against the broth microdilution reference are also presented for each isolate; True Negative (TN), True Positive (TP), False Negative (FN) and False Positive (FP). **Table S3.** presents European Read Archive run accession numbers for each sequences isolate. **Table S4.** presents the frequency of isolates with each reference resistance gene sequence detected or fully assembled by ARIBA. (XLSX 144 kb)

## Data Availability

Whole genome sequencing data is available in the NCBI Short Read Archive under BioProject accession PRJEB10999. Run accession numbers are listed per-sample in Additional file 1: Table S3. All other data generated or analysed during this study are included in this published article and its supplementary information files.

## Abbreviations

AMR: Antimicrobial resistance
AST: Antimicrobial susceptibility tests
ECOFFs: Epidemiological cut-offs
EUCAST: European Committee on Antimicrobial Susceptibility testing
ME: Major error rates
MIC: Minimum inhibitory concentration
VME: Very major error rates
WGS: Whole genome sequencing (WGS)
